# P01-026 – A case of FMF and hereditary coproporphyria

**DOI:** 10.1186/1546-0096-11-S1-A30

**Published:** 2013-11-08

**Authors:** A Ganesha, S Savic

**Affiliations:** 1 Clinical Immunology and Allergy, St James's University Hospital, Leeds, United Kingdom

## Introduction

We report a unique case in a 17 year old male patient of Algerian origin with two rare genetic conditions with overlapping clinical symptoms. Familial Mediterranean fever (FMF) is an autosomal recessive disorder characterized by sporadic, paroxysmal attacks of fever and serositis. Hereditary coproporphyria (HCP) is one of the type of acute hepatic porphyria resulting in neurovisceral symptoms caused by deficient activity of mitochondrial enzyme coproporphyrinogen oxidase. Both are considered rare differential diagnosis for acute abdominal pain.

## Case report

17 year old boy of Algerian origin presented with long history of recurrent episodes of fever, abdominal pain since infancy. He experiencied 3-4 attacks per year each lasting typically for 2-3 days. There was no family history.

Patient was referred simultaneously to immunology and metabolic medicine for further assessment. Differential diagnoses considered at the time included: periodic fever syndromes, hereditary angioedema, vasculitis and porphyria.

FBC results over the year showed intermittent leucocytosis during acute attacks with elevated C-reactive protein (CRP) and plasma viscosity (PV). Serum amyloid A (SAA) was not measured. Investigations during quiescent phase showed normal levels of SAA but slightly elevated CRP 13.4 mg/l (ref <10) and neutrophilia of 9.9 x10^9^/l (ref 2.00-7.50).

Genetic investigations for periodic fever syndromes confirmed two pathogenic MEFV gene mutation on sequencing Exon 2 and 10 at p. (Met694IIe(;)Glu148Gln), supporting diagnoses of FMF. Sequencing for MVK and TNFRSF1A gene were negative.

Investigations undertaken by metabolic medicine specialists revealed urine coproporphyrin III at 42.75nmol/mmol cret (1.2-24.8) with porphrin/creat Ratio of 54.6 nmol/mmol (Ref <28). Faecal porphrin were 1639nmol/g dry weight (ref <130) with faecal coproporphyrin III: I ratio at 19.51 (Ref <2). The results confirmed diagnosis of HCP. Genetic tests are awaited for the patient. He has management plan for hereditary coproporphyria.

Patient was commenced on colchicine at the dose of 500 micrograms twice daily. No further episodes of abdominal pain have been reported in the last 9 months since prophylaxis starting prophylaxis with colchicine.

## Discussion

FMF and HCP are both recognised as rare causes of unexplained acute abdominal pain associated with fever. However there are several additional features which would favour FMF over HCP. The patient’s ethic origin is more suggestive of FMF. The majority of HPC was reported in the North European ancestry. The onset of HCP also tends to be in puberty whilst FMF usually presents in childhood with initial attacks before the age of 10 in 65% of cases. Low grade persistent inflammatory response is a feature of FMF and not necessarily seen HCP. Finally apparent response to colchicine would further support diagnosis of FMF.

## Disclosure of interest

None declared.

## References

[B1] Tunca M Familial Mediterranean fever (FMF) in Turkey: results of a nationwide multicenter study Medicine (Baltimore) 2005 11 1 1 15643295 10.1097/01.md.0000152370.84628.0c

[B2] With TK Hereditary coproporphyria and variegate porphyria in Denmark Dan Med Bull 1983 11 2 106 6851678

